# In Vitro Evaluation of the Effect of Stenting on Hematological, Hemorheological and Hemodynamic Parameters, in Various Stent Configurations and Flow Conditions

**DOI:** 10.1007/s13239-025-00791-0

**Published:** 2025-06-25

**Authors:** D. Kokkinidou, K. Kapnisis, M. Chrysostomou, C. Shammas, A. Anayiotos, E. Kaliviotis

**Affiliations:** 1 Department of Mechanical Engineering and Materials Science and Engineering, Cyprus University of Technology, Limassol, Cyprus; 2 Bioanalysis Clinical Laboratory, Spyrou Kyprianou 23C, Limassol, Cyprus

**Keywords:** Stents, Stent-induced changes, Red blood cell biomechanics, Hemorheology, Hematology, Hemodynamics, Pressure-drop

## Abstract

**Purpose:**

Percutaneous coronary intervention is used extensively for the restoration of blood flow in diseased arteries. The influence of stent implantation on the physiology and flow of blood is an important and still not fully understood issue. The current work evaluated possible stent-induced changes in hematological, hemorheological and hemodynamic parameters.

**Methods:**

Experiments were performed for blood flow in single and overlapping stent configurations, in both straight and curved tube geometries, in order to reproduce various stented coronary artery morphologies. Two different flow regimes were utilized to reflect a range of physiological and more intense flow conditions. Blood samples were obtained from a healthy human population and commercially available stents were inserted in clear perfluoroalkoxy alkane tubing, connected to a syringe/syringe-pump/pressure-sensor setup. Hematological measurements, red blood cell (RBC) deformability and aggregation, and whole blood viscosity tests were performed using standard techniques. The pressure drop across the stented area was measured via an in-line pressure sensing setup.

**Results:**

In terms of hematology, RBC count, hematocrit, and mean corpuscular volume show a slight influence from the longer exposure to elevated stresses. Regarding hemorheology, the most profound effect was observed on RBC aggregation, with an increasing trend primarily in the female population of the study. Further, differences were found in the hemodynamics of the flow, as the pressure drop was altered according to the stent configuration. The viscosity of the blood samples is also found affected in the higher flow rate cases.

**Conclusions:**

The presence of the stent was found to have a distinct effect on specific hemorheological and hemodynamic parameters according to the setup and stent configuration.

## Introduction

Percutaneous coronary intervention (PCI) is a non-surgical procedure, which is performed to open blocked heart arteries with the use of appropriate stents [1]. Thrombogenicity and restenosis after stent placement are influenced by several factors, including stent design characteristics [2], with strut thickness and density the most important design factors affecting thrombogenicity [3]. Stent underexpansion and malapposition have also been linked to a high risk of in-stent restenosis and thrombosis [4, 5].

However, while stent design parameters contribute to thrombogenicity, the pathophysiological progression of atherosclerosis remains a key determinant of very late stent thrombosis. Thrombus formation according to Virchow’s triad, is driven by stasis of blood flow, endothelial injury and hypercoagulability [6]. A contributing factor is the development of de novo atherosclerotic lesions in the proximal segment of a stented artery. As the lesion develops, the progressive luminal narrowing disrupts normal blood flow dynamics, leading to stasis in the distal stented region. Additionally, myocardial bridging can disrupt endothelial function, with the associated turbulent shear stress and intimal damage, increasing the vessel’s susceptibility to thrombus formation [7].

Stent implantation alters local hemodynamics and other hemorheological parameters [8]. In vivo studies investigating the effects of stenting on rheological parameters indicate an initial reduction in whole blood and plasma viscosity immediately following stent placement. This decrease in viscosity is subsequently followed by a significant increase within a few days and a notable decrease approximately one month post-implantation [9, 10], suggesting that such rheological variations may play a role in early stent occlusion. In an in vivo study, it was demonstrated that the presence of a stent within a tube alters specific rheological properties of blood under high-flow conditions [11]. Notably, elevated viscosity was observed when blood experienced prolonged exposure to increased flow rates, accompanied by decreased aggregation and deformability. Kokkinidou et al. [12], investigated the effects of stenting and implantation duration on different hematological and hemorheological parameters in male CD1 mice. Custom nitinol stents were implanted in the common carotid artery, and hematological assessments were conducted at two different time points, at 5 and 10 weeks post-implantation. Results revealed possible implant-induced alterations in hematological and hemorheological indices, including increased blood viscosity and reduced red blood cell deformability. In addition, Baars et al. [13], demonstrated that stent implantation into atherosclerotic coronary arteries may lead to increased red blood cell aggregation, which could potentially compromise microvascular perfusion. These findings point out the need for further investigation, in order to elucidate the physiological implications of hemorheological changes associated with cardiovascular stenting.

Another hemodynamic aspect of interest in the stented region is the pressure drop across the implant. In the computational study of Ahadi et al [14], the effect of stenting was investigated by using Newtonian and non-Newtonian models for blood viscosity. The highest pressure drop, compared to the non-stented flow case, was observed with the Carreau viscosity model, and it was 10% more than the result of the Newtonian flow case. A power-law viscosity model showed the lowest pressure drop, which was approximately 2.3% less than the Newtonian model [14].

In another CFD study by Gamage et al. [15], it was shown that the vessel outlet pressure increased after stenting, with the post-dilation of the implanted stent, reducing the pressure drop across the vessel. Specifically, the pressure drop decreased from 24.58 to 19.96 mmHg after stenting, and further decreased to 18.46 and 16.02 mmHg following two post-dilation procedures (at 20 and 30 atm balloon pressures respectively). Another study by Koskinas et al. [16] reported that regions experiencing high shear stress due to stent placement are more prone to endothelial (tissue) injury and platelet aggregation, both of which are factors contributing to restenosis.

The stiffness of the stented vessel region due to stent rigidity has also various hemodynamic effects. Alderson and Zamir [17], investigated this issue analytically and showed that the stent placed in a larger diseased vessel creates higher pressure at the entrance compared to the exit. This may occur because the stent’s relative rigidity reduces wave propagation within the vessel. More specifically, wave reflections that return to the vessel entrance cause an increase in pressure in that location, while the waves that pass through the stent have a reduced amplitude due to the damping effect of the stent.

The present study builds up and expands on previous knowledge by thoroughly investigating in an ex vivo setting, stent-induced changes in hematological hemodynamic and hemorheological parameters. The study covers single and overlapping stent cases, to account for common clinical practices, in both straight and curved modes to reproduce various coronary artery morphologies in the cardiac cycle, and under two different biomechanical flow regimes to reflect a range of physiological flow conditions.

## Materials and Methods

### Blood Sample Preparation

The study protocol was approved and granted by the Cyprus National Bioethics Committee (ref: EEBK/E/2016/18). Blood samples (30 ml) were obtained from a healthy population (8 volunteers, equal number of male and female; aged 20–50 years) using a 21G needle and collected into 9 ml vacuum tubes (BD) containing 1.8 mg/ml EDTA. All tubes were then placed in a blood mixer device for 2–3 min, to ensure proper mixing with the anticoagulant. Before and during each step of the procedure, the blood samples were carefully visually examined to assess for any signs of sedimentation or abnormalities. All blood samples were transferred in a 50 ml syringe and oxygenated by rolling carefully for approximately 1/2 minute, before extracting the air from the syringe. Testing was performed immediately after blood withdrawal.

### Stents Design and Tube Stenting Configuration

Commercially available stents were inserted in clear perfluoroalkoxy alkane (PFA) tubing of internal diameter 2.5 mm in single and overlapping (~ 30%) configurations. The single stented case included a Boston Scientific stent (PROMUS Element platinum-chromium (PtCr)) with dimensions 3 × 8 mm (nominal diameter × length) and a strut thickness of 93 μm (including polymer coating). For the overlapping case, two stents of similar geometry and dimensional characteristic cells were used: a Boston Scientific PROMUS Element stent (3.5 × 32 mm − strut thickness 93 μm) and an Abbott Xience PRIME cobalt-chromium (CoCr) stent (2.75 × 28 mm − strut thickness of 97 μm, including the polymer coating).

Stents were inflated at a predetermined balloon pressure (10–12 atm as per the manufacturer’s guidelines) to achieve optimal expansion with a stent-to-tube-size ratio of ~ 1.1. Stent apposition and stability were assessed under a stereomicroscope and confirmed by subjecting the stented tube to a very high flow rate (50% higher than the maximum flow rate applied during testing). Identical but empty tube setups were employed as the control cases. All three configurations (control, single, and overlapped stented) were tested in two geometries, straight and curved (radius of curvature = 20 mm). These configurations were chosen to replicate coronary artery morphologies observed in the cardiac circulatory system. The radius of curvature was chosen based on the work of Wei et al. [18], and Forouzandeh et al. [19] which investigated different curvatures (radii of 64 mm, 40 mm, and 16 mm) to represent a range of physiologically relevant arterial geometries. These values were derived from existing computational and experimental studies, ensuring that the modeled geometries encompass a broad spectrum of realistic coronary artery curvatures.

### Experimental Setup and Procedure Protocol

**Fig. 1:**
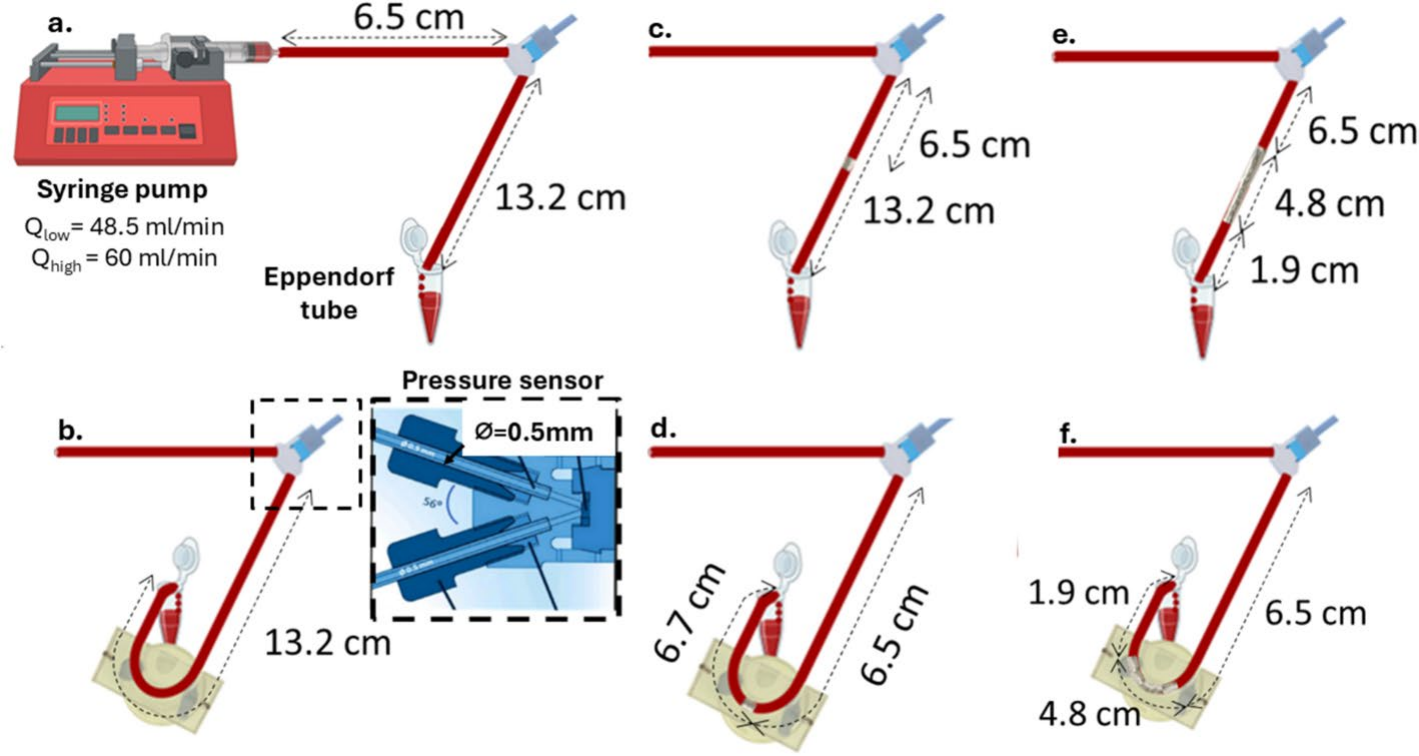
Schematic illustration of the experimental design. Experimental cases include setups for: **a** straight non-stented case, **b** curved non-stented case, **c** single stented straight case, **d** single stented curved case, **e** overlapped stents in a straight tube, and **f** overlapped stents in a curved tube

The experimental setup, illustrating the six different cases, is shown in Fig. 1. The 50 ml syringe, containing the blood sample, was placed on a syringe pump (KDS 200) and was linked to a pressure sensor through an empty 2.5 mm diameter PFA tubing of 6.5 mm length. A second (test) tube, containing the stent samples, was linked to the other end of the pressure sensor, and the perfused blood specimens were collected within a 2 ml Eppendorf tube (see Fig. 1). The end of the tubing was in contact with the Eppendorf tube wall for smooth sliding flow to avoid splashing. The test tube had a length of 13.2 mm with the stent(s) placed in the middle. To ensure a fully developed flow in the region of the pressure sensor and stent, the entrance length (L) was calculated based on the relationship between the length-to-diameter ratio $L/d$ and the Reynolds number (Re): $L/d= 0.06*Re$.

The Reynolds number was estimated using the high shear viscosity of blood at 5 mPas. The lengths of both the tubes used were several times larger than the entrance lengths. The pressure monitoring setup consisted of an Elveflow OB1 MK3 pressure controller (Elveflow, Paris, France) connected with the Elvesys inline microfluidic pressure sensor (MPS3, − 1 to + 1 bar range, Elveflow, Paris, France). The pressure sensor was calibrated before each test to ensure accurate pressure readings, and data were continuously recorded with a 10 Hz acquisition rate for the duration of the flow.

Two different flow regimes were adopted to reflect a range of physiological flow conditions (systolic/diastolic peak and mean flow values) of the left anterior descending coronary artery [20]. The first was a baseline, low-exposure regime, where blood samples were exposed to a single passage through the stented tube, with a flow rate of 48.5 ml/min. In a second regime, higher stress and longer time exposure conditions were set, utilizing a quadruplicate blood passage (back-and-forth flow through the stented tube) with a flow rate of 60 ml/min. In both cases, the sequence of blood flow experiments adhered to the following order: (1) flow in the straight, non-stented tube (control straight), (2) flow in the curved non-stented tube (control curved), (3) flow within the single stented tube (straight and curved tube) and (4) flow within the overlapped stents (straight and curved tube). After each test, the stented tube was thoroughly cleaned by infusing/withdrawing distilled water multiple (×6) times, followed by high-flow air infusion for 30 secs in order to fully dry the tube and stent. Stents were inspected again visually for cleanness and integrity before undertaking the next experiment. Additionally, during each setup change, the 50 ml syringe along with the linked tubing was carefully removed and subjected to a rolling motion to prevent red blood cell (RBC) sedimentation.

### Hematology

Hematological testing was completed within a 3-hour timespan, in a qualified Hematology Laboratory. The Sysmex XT-2000i analyzer (Sysmex, Landskrona, Sweden) was utilized for hematological measurements which in all blood samples included white blood cell (WBC) and red blood cell (RBC) counts, hematocrit (HCT), mean corpuscular volume (MCV), and red blood cell distribution width-coefficient of variation (RDW-CV).

### Hemorheology and Hemodynamics

The pressure drop between the sensor and the open end of the test tubes (atmospheric conditions) was continuously recorded with a 10 Hz acquisition rate for the duration of the flow. The pressure monitoring setup consisted of an Elveflow OB1 MK3 pressure controller (Elveflow, Paris, France) connected with the Elvesys inline microfluidic pressure sensor (MPS3, − 1 to + 1 bar range, Elveflow, Paris, France). The pressure drop results are expressed as the mean of the maximum pressure values of the two repetitions ($\overline{\Delta P}$) in the perfusing mode (pushing part in the case of the reciprocating flows) and for all volunteers ($\overline{\overline{\Delta P}}$ with n = 7).

Hemorheological evaluation commenced immediately after flow testing. The hemorheological analyses included blood viscosity, red blood cell aggregation, and deformability. All the hemorheological parameters were assessed using standard equipment and methodologies at a controlled room temperature (25 ± 0.5 °C). The viscosity of each sample was measured using the cone-plate Brookfield DV2TLV instrument (AMETEK GB LTD T/A Brookfield, Stadium Way Harlow, Essex). All blood measurements were conducted at the same predetermined shear rates, ranging from 251.2 to 0.98 s⁻^1^ (from higher to lower shear rates to ensure initial dispersion of aggregates). Viscosity data were included in the analysis for transducer torque values greater than 5% of the total torque range. Normalized viscosity was calculated as ${\eta }^{*}=\frac{{\eta }_{i}}{{\eta }_{c}}$, where c represents the control configuration blood viscosity and $i$ represents the viscosity measured for the blood samples flowed in the different stented tube configurations. Further, the viscosity data were normalized with their high shear rate values (at 251.2 s^−1^) for each case, in order to focus the analysis on the non-Newtonian viscosity behavior, which is affected by both RBC aggregation and deformability.

The Rheoscan A200 instrument (Rheomeditech, Seoul, Korea) was employed to assess the red blood cell aggregation of the various samples. The aggregation index (AI was utilized as a metric to evaluate the overall extent of RBC aggregation. AI is defined as the ratio of the areas above and below a time-dependent curve (a laser backscattering signal), produced by the application of a laser source on a microchip containing the blood sample [21]. Another useful aggregation index used to verify the resulting AI behavior is the aggregation half-time T_1/2_ index, which indicates the time to reach half of the aggregation potential. RBC deformability measurements were determined using the Rheoscan D300 (Rheomeditech, Seoul, Korea). The instrument employs a laser diffraction technique applied on a disposable microfluidic chip [22]. In this setup, blood is perfused through the microfluidic chip at varying pressures, inducing ellipsoidal deformation of the cells. The cells’ elongation index (EI), is defined from the major (A) and minor (B) lengths of the ellipsoidal axes of the deformed RBC as (A-B)/(A + B). EI is then calculated across various shear stresses (SS) ranging between 0 and 20 Pa. The maximum EI (EImax) was selected as the representative deformability parameter to facilitate comparison between samples.

### Statistical Analysis

A two-way ANOVA test was employed to compare the hematological and hemorheological variations among the six groups of samples for each condition (normal/higher exposure) and for each stent geometry/configuration investigated in the study. In contrast, one-way ANOVA was used for pressure drop comparisons with one independent variable. The Kolmogorov-Smirnov test was employed for the normality check. All measurements were presented as mean ± standard deviation (SD), and the level of significance was considered at p < 0.05.

## Results

### Hematological Evaluation

**Fig. 2:**
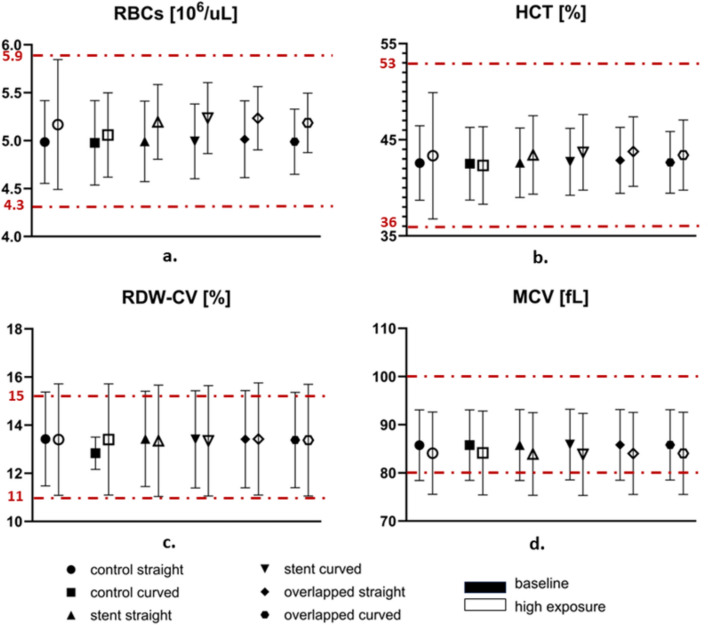
Effect of stenting on various hematological parameters for the different experimental setups and the different exposure regimes. **a** RBC count, **b** Hematocrit, **c** RBC width distribution, and **d** Mean corpuscular volume. Values are expressed as the mean ± SD. The physiological range for each parameter is indicated by the red dashed lines

Figure 2 presents the hematological indices for the blood samples tested in the different stenting cases and different flow conditions. The results are presented as mean values from all tests performed (n = 8) with their standard deviations placed as error bars. Along with the results, the physiological range for each parameter is indicated with the red dashed lines. Although no statistically significant differences were detected between the twelve tested conditions, some noticeable tendencies are present, which could be attributed to tube stenting and the geometric configurations used. These include the hematological changes between the baseline (low-exposure regime) and the high-exposure conditions. More specifically, an increase in RBC count and hematocrit (HCT) levels is observed (Fig. 2a and b), accompanied by a concurrent decrease in the mean corpuscular volume (MCV, in panel 2d) at high-exposure conditions compared to the baseline. These findings could also account for the higher RDW-CV (Fig. 2c) in each case, which is found elevated in the high-exposure regime.

In general, the differences between the two exposure regimes seem to be systematic for all utilized setups, except for the control-curved configuration. Another observation made from Fig. 2, is that the SD in RDW-CV reaches the physiological range, while those of the MCV extend below the physiological range.

Considering the high-exposure data, it appears that there is an increasing tendency (although not significant) in the RBC count, between the control (curved) and the stented cases. In the absence of physiological factors, this increase in the RBC count could only be attributed to known flow parameters, such as alteration of the hematocrit profile in the tube, resulting in a higher concentration of cells in the bulk [23].

### Hemorheology and Hemodynamics

**Fig. 3:**
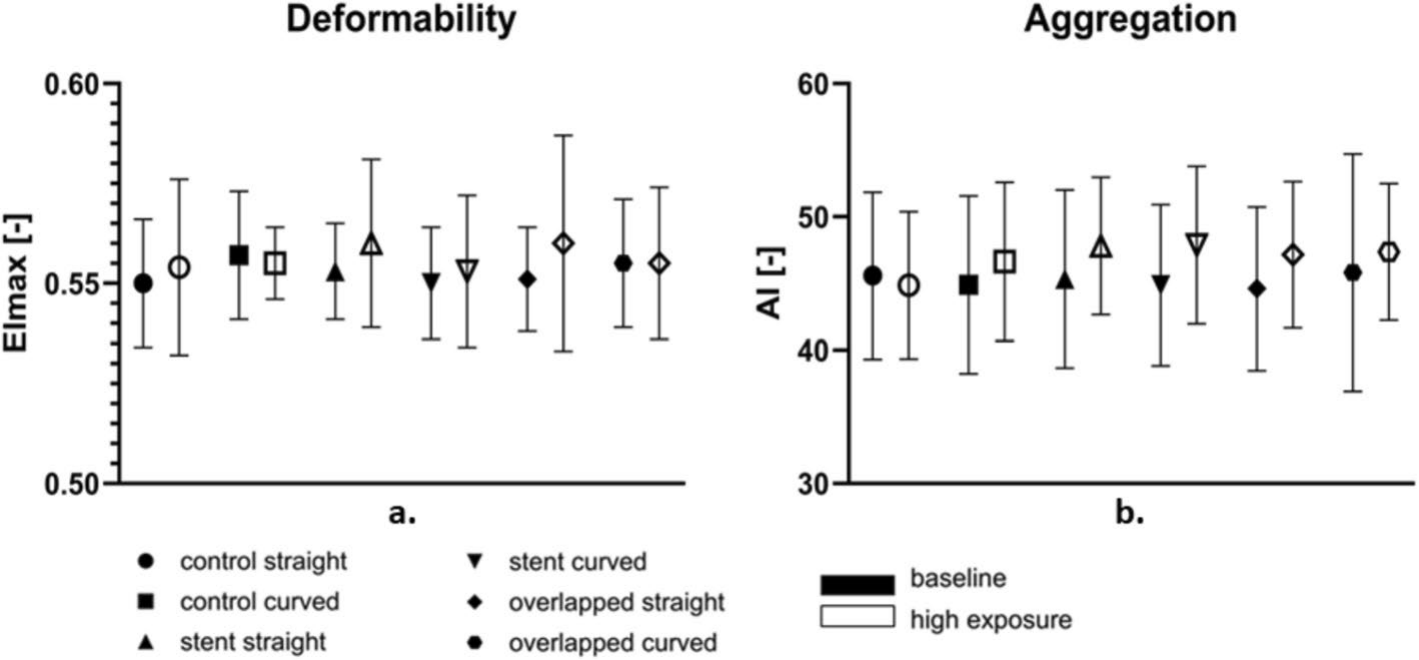
Effect of different exposure conditions (baseline vs high-exposure) in RBC deformability and aggregation

The hemorheological parameters RBC deformability and aggregation are expressed by the indices EI_max_ and AI respectively, and are presented in Fig. 3. The figure compares the results from the low-exposure tests (baseline tests at the lower flow rate, and lower time exposure) with those from the higher flow and time exposure in the reciprocating mode. Figure 3 shows a slightly higher-than-normal deformability for all samples and test modes, as normal values of EI_max_ are found in the vicinity of 0.5. This increase can be attributed to factors such as tourniquet application [24] for blood collection, as it has been found that this approach can result in approximately 8% higher than the control values measured in samples obtained without a tourniquet use [24].

Furthermore, Fig. 3 illustrates that EI_max_ is increased in the high-exposure cases compared to the low-exposure baseline regime, for all experimental configurations. The same increasing tendency was observed in the AI. This could be explained by the elevated flow and stress conditions and time exposure that cells experience in the particular flow mode. More specifically, the increased flow rate utilized in the high-exposure regime seems to result in increased deformability and aggregation. Regarding the latter observation, it is known from the literature that the time duration the RBCs are exposed to stresses has a non-monotonic effect on the elastic properties of the cells [25]. Thus, according to shear stress and time exposure, the deformability of the RBCs can be affected positively or negatively, and the effect can be either permanent or temporary. Another study has shown that the kinetics of RBC aggregation are increased for cells with increased deformability [26], suggesting a similar effect in the present study.

**Fig. 4:**
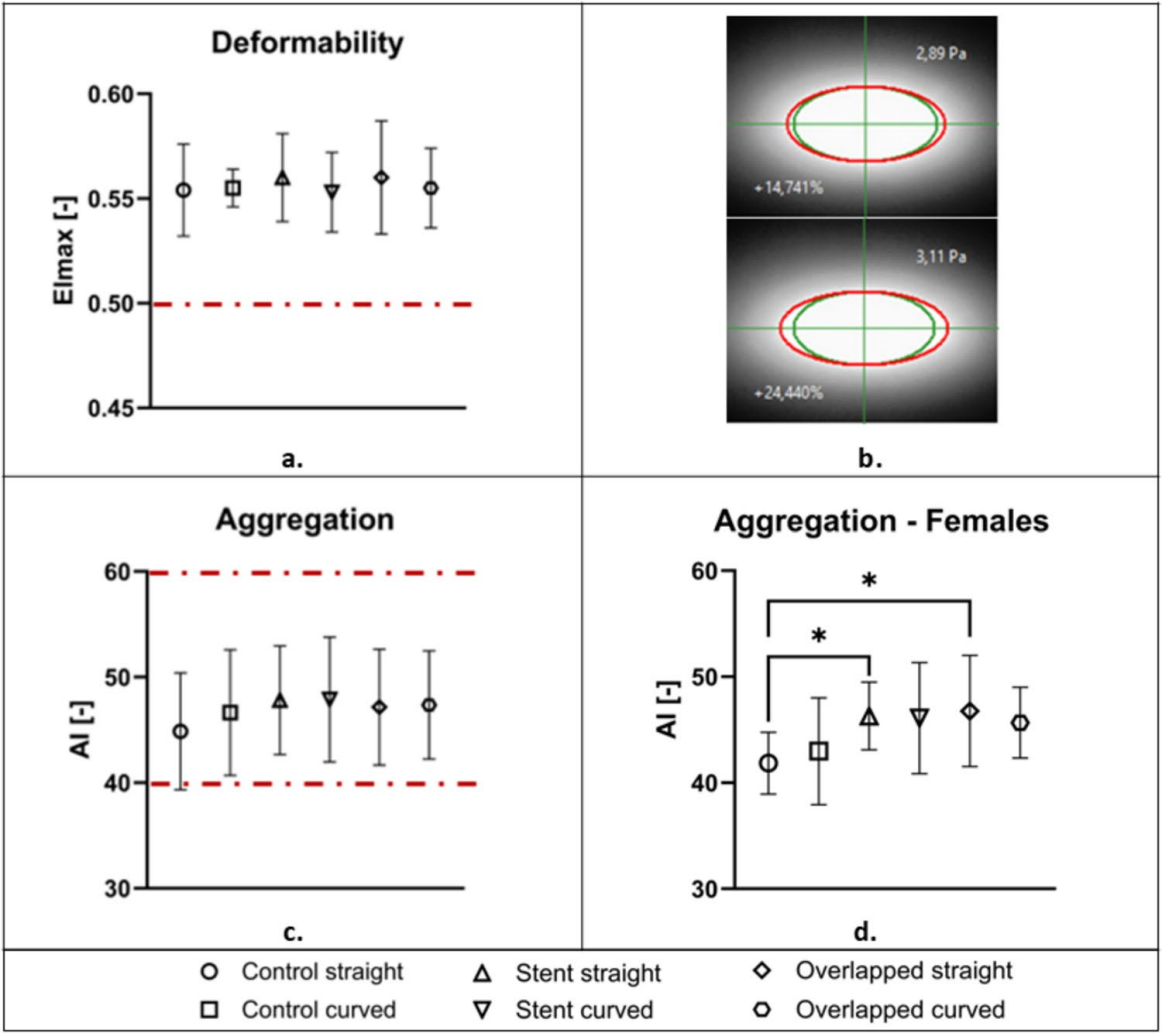
Effect of stent and geometry for the high-exposure conditions in RBC deformability (**a**) and aggregation (**c**). The physiological values for each parameter are indicated by the red dashed lines. **b** Images of the diffraction pattern resulting from the deformed RBCs at comparable shear stresses (2.89 and 3.11 Pa) for the Control Straight (top image) and the Overlapped Curved (bottom image). In green color, the expected physiological elliptical pattern for the specific stresses. The percent differences from the physiological pattern are given as the difference in the ellipse area for each case. **d** AI index for the female population of the study (*for p < 0.05; two-way ANOVA)

The small alteration in RBC deformability and aggregation between the test configurations of the high-exposure condition are illustrated more clearly in Fig. 4. A slight decrease in the deformability index is observed in Fig. 4a between the cases of single-stent from straight to curved geometries. Similarly, for the cases of overlapped stents, from straight to curved geometries. These small differences, however, do not show any statistical significance.

Images of the deformed pattern of RBCs, as extracted from the Rheoscan-D300 instrument, for two comparable shear stresses (2.89 and 3.11 Pa) and two configurations (control straight and overlapped curved) are shown in Fig. 4b. In green color are the expected physiological elliptical patterns for the specific stresses. The percent difference from the physiological pattern is also given as the difference in the ellipse area for each case.

The aggregation index shown in Fig. 4c seems unchanged for the abovementioned cases (stented and overlapping), however, AI is found to be increased in all the stented samples compared to the control cases, and also when comparing the control-straight configuration, to the control-curved case. These findings imply that the stent presence within a vessel may have a small, albeit noticeable, effect on the aforementioned hemorheological properties of blood. This tendency of RBC aggregation to be increased in the stented cases was found to be mainly due to the female participants of the study (n = 4). Figure 4d shows that statistically significant differences are found between the Control Straight and the straight Stented and Overlapping cases (p < 0.05).

**Fig. 5:**
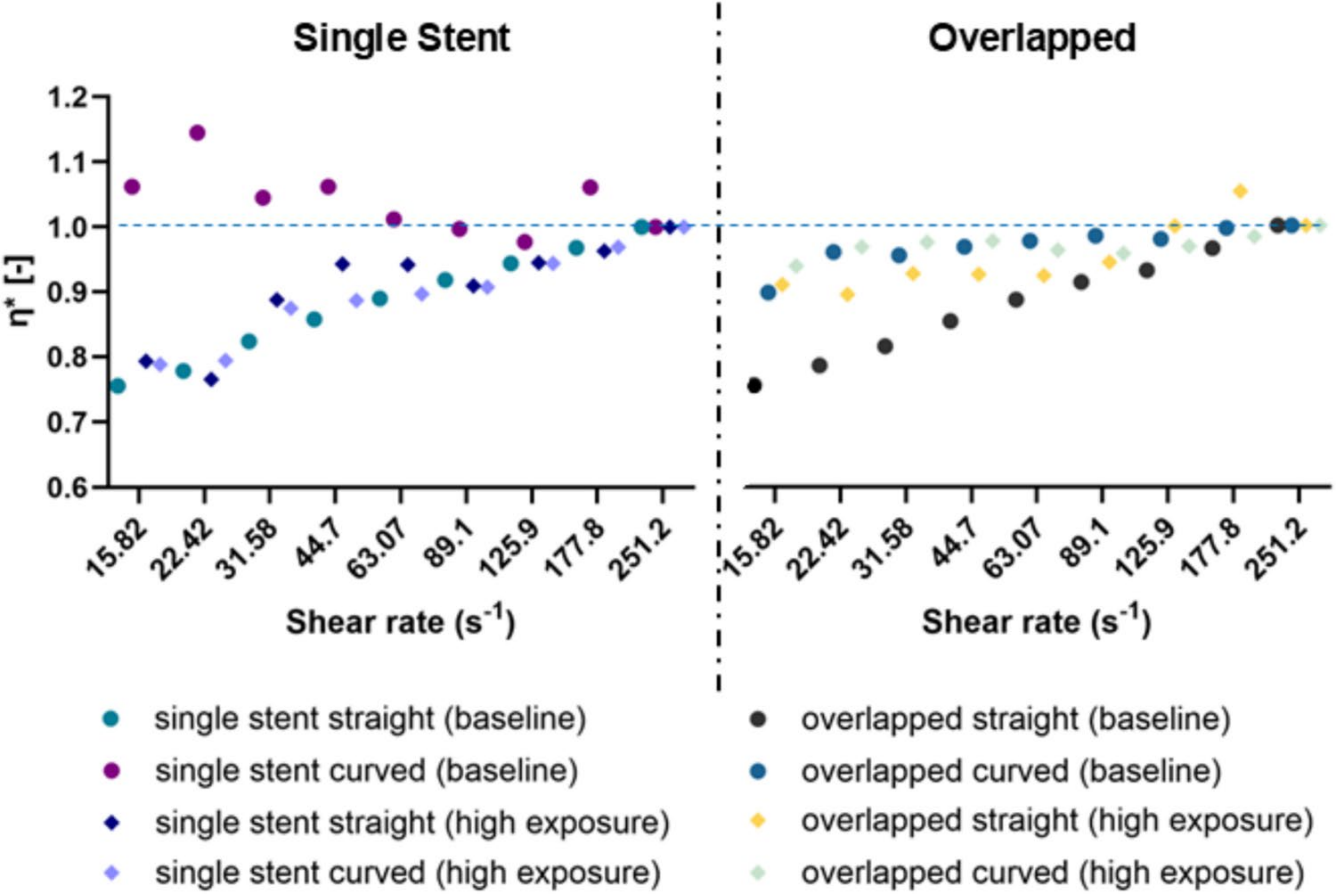
Viscosity alterations η* in single stented and overlapped cases

The normalized (η*) viscosity data, grouped for the single-stented and overlapping stent setups, are shown in Fig. 5, for a shear rate range of 15.82 to 252.2 s^−1^. The general observation from Fig. 5 is that, in most cases, the non-Newtonian part for all configurations, and in both exposure regimes is suppressed compared to the control cases. This decrease in viscosity could be attributed to geometry and configuration effects on the hemorheological parameters discussed earlier. The slight increase in deformability, seen in Fig. 4 for the stented cases collectively, may have positively affected the viscosity of the samples. However, the increase in HCT and RBC aggregation (see Figs. 2 and 4), although very small, is expected to counterbalance the influence of deformability and negatively affect the viscosity of the samples.

As regards the viscosity differences between the various configurations, it can be observed from the overlapped-stent cases in Fig. 5, that the viscosity is more profoundly closer to the control cases for the curved geometry samples, which could be linked to the increased RBC aggregation seen in those cases (see Fig. 4). Nevertheless, no statistical significance is observed for the η* differences between the samples.

**Fig. 6:**
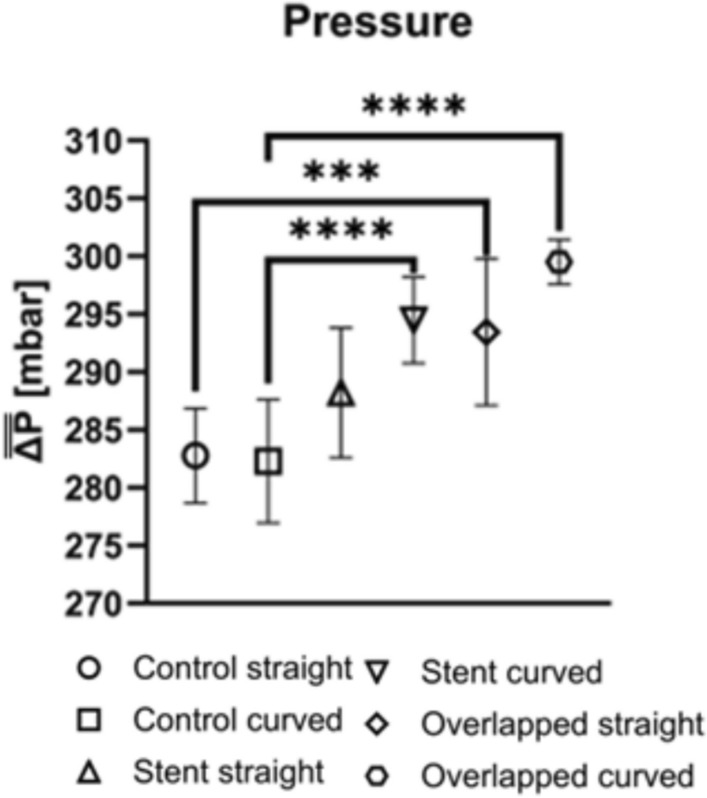
Mean Pressure $\overline{\overline{\Delta P}}$ from all volunteers for every stent configuration. (*** for p < 0.001; **** for p < 0.0001; one-way ANOVA)

Results for the pressure drop across the stented area are shown in Fig. 6. $\overline{\overline{\Delta P}}$ is expressed as a mean value for the two perfusion parts in the reciprocating mode, averaged for all volunteers (n = 7) in the high-exposure tests. Figure 6 designates the existing trends between the control cases and the different stenting configurations. A statistically significant difference in $\overline{\overline{\Delta P}}$ (p < 0.001) was observed between the control straight and the overlapped straight stenting configurations. In general, the overlapping stent setups resulted in a greater $\overline{\overline{\Delta P}}$ value compared to the control groups, suggesting that stent overlapping and tube curvature have a measurable effect on the local pressure developed inside the tube. When comparing the curved configurations between them, an additional statistically significant difference (p < 0.0001) is observed between the control and the stented (single and overlapped) configurations. This suggests that the curved geometry, along with further flow obstacles (overlapped stent), affects the pressure drop differently than the single stent configuration.

No statistically significant difference in $\overline{\overline{\Delta P}}$ was found between the control cases and the single stent configurations when grouped together, suggesting a small effect of the latter on the hemodynamics of the flow.

## Discussion

### Hematology Effects

The results of the present study showed an observable increasing tendency between baseline and high-exposure conditions, mainly on RBC and HCT. Simultaneously, MCV seems reduced after exposure to high compared to baseline conditions. This pattern is in agreement with previous studies, indicating that mechanical stresses and altered flow dynamics, may impact RBC morphology and volume [27]. In human biology, rising RBC count and HCT indicate a physiological response to the altered hemodynamic environment, potentially functioning as an adaptive mechanism to maintain adequate oxygen delivery under varying shear stress conditions [28]. HCT is directly linked to the RBC mechanism of erythropoietin, which stimulates the bone marrow to produce mature RBCs, leading to an increase in hematocrit levels [29]. In the in vivo model of the present study, however, the increase in RBC count and HCT could only be attributed to fluid mechanics and experimental factors. Factors such as the shear stress generated by fluid flow, the geometry of the experimental setup, and the blood handling procedures, can impact the results [30]. The MCV reduction seen in Fig. 2 could be explained by the increased flow rate and repeated passage in the high-exposure case, resulting in RBC cells becoming more compact and deformable. Wiegmann et al. [30], observed that MCV decreases after exposure to high pressures. The combination of increased RBC and decreased MCV may have led to the observed increase in RDW-CV.

### Hemorheological Parameters

Experimental evidence has established the concept of active regulation of RBC deformability, which is primarily associated with altered interactions between membrane skeletal proteins and the integral proteins of the lipid bilayer. Consequently, it has been hypothesized that shear stress induces changes in RBC deformability; Meram et al. [31], investigated the transient improvement in RBC deformability using a Couette-type shearing system and showed that increased levels of shear stress (within 5-20 Pa) improved tissue perfusion by enhancing nitric oxide (NO) production by endothelial cells. Simmonds et al. [32], demonstrated that by applying consistent mechanical stress within the physiological range of 5–20 Pa, RBC deformability can be improved. However, it should be noted that shear stress can also impact circulating platelets, causing their activation and aggregation and thereby increasing the risk of thrombosis [31]. In the present study, the maximum shear stress is expected to appear in the region of the stent, where the flow dimensions decrease further, and the geometry becomes complex. For a steady and fully developed flow of a non-Newtonian (Power-Law) fluid in a uniform geometry, the maximum shear stress at the walls is estimated to reach approximately 4 Pa. Therefore, moderate stress levels are expected to affect the deformability of RBCs accordingly.

Blood oxygenation may be another parameter affecting the deformability of RBCs. The effects of oxygenation on erythrocyte flow velocity and shear-induced deformability were assessed through microfluidic flows, with different channel dimensions (5 μm, 20 μm) [28]. Enhanced RBC deformability was associated with enhanced erythrocyte velocity in a microchannel flow, resulting however, in a reduced oxygen supply. In the present work, the fluid-air interface at the open front of the flow could introduce blood re-oxygenation, however, this is expected to have a small and systematic effect on all samples and cases tested. Since the deformability seems altered in the higher, compared to the low-exposure mode, it could be argued that oxygen supply could be inhibited in an In vivo situation due to the velocity in the arterioles and capillaries being increased.

RBC membrane properties, such as cytoskeleton elasticity, and the viscosity of the cytoplasm contribute to the speed and degree of membrane relaxation, which denotes the return to the normal cell shape after deformation. It has been shown that the phosphorylation of beta spectrin by Casein kinase II, and protein kinase C-catalysed phosphorylation of protein 4.1, are correlated with elevated RBC deformability through specific receptors. The mechanical properties of blood are also affected by the fluctuations of serine phosphorylation of spectrin [33]. In the present work, any effects of stress exposure on the biochemistry of the RBC membrane could not be accounted for and perhaps will be the subject of another study.

RBC aggregation is another parameter affecting blood rheology and hemodynamics of the tube flow. In the in vivo setup, increased RBC aggregation has been associated with decreased flow resistance in vertical capillaries, while increased flow resistance is observed in horizontal capillaries [34]. Another similar study, on experiments conducted in vertical and horizontal glass tubes, revealed that in horizontal tubes, cell sedimentation on the lower wall increases hydrodynamic resistance, forming a nearly stagnant cell mass [35]. Plasma flows over this mass, entraining corpuscles from the upper layers. Knisely et al. [36], termed this phenomenon "sludging" and attributed it solely to pathological conditions, noting its absence in healthy individuals and animals [35]. Nader et al. [37], showed that RBC aggregates can persist in large arteries and affect flow dynamics [37]. Further, increased RBC aggregation promotes RBC axial migration, increasing the cell-free layer width, which in turn decreases the wall shear stresses, NO production, vasodilation, and blood viscosity [23, 37]. The aforementioned RBC-aggregation-related phenomena are intense mostly in low flow rates, and therefore low shear rates. In the present study, blood was subjected to moderate to higher shear rates, and such effects are expected not to influence the flow significantly. This is also apparent in Fig. 4, showing normal aggregation levels. The statistically significant differences in the RBC aggregation observed in the present study in the female population, may indicate a potential influence of the stent on the phenomenon, however, all values remain within the physiological levels.

RBCs are the main determinant of blood viscosity and therefore affect the frictional forces exerted by the circulating blood on the arterial wall [38]. The results in the present study indicate that stenting may have a tracible positive influence on the viscosity of the fluids and that this may be the result of changes in the RBC properties. More specifically, the non-Newtonian behavior of blood viscosity measured for the samples of the stented configurations collectively seems to be reduced at low shear rates, when compared to the control cases (in both baseline and high-exposure cases in Fig. 5). This could be attributed to the improved deformability of the RBCs, observed between the control and the stented cases (see Figs. 3a and 4a). On the other hand, RBC aggregation seems to be slightly increased in the high-exposure stented cases, compared to their control samples (Figs. 3b and 4b). As mentioned earlier this increase in RBC aggregation might be the result of altered deformability. As related studies have indicated, the elongation index EI_max_ and RBC shape recovery time are positively correlated with the aggregation index AI [26].

The hematocrit of the samples in the baseline and high-exposure cases, seem largely unaltered (Fig. 2). In the high-exposure cases, a slight increase could be detected for the stented configurations when compared collectively to the control cases (38.90 and 38.15 respectively), however, this difference is considered to be insignificant and therefore has a negligible effect on the viscosity of the samples.

### Local Hemodynamics

Local geometrical characteristics of the stented area are expected to influence the local flow in a complex manner [11]. For instance, secondary frictional losses due to diameter reduction in the stent area and curvature of the tube are expected to influence the total pressure drop in the tube. The results of the present study illustrate the impact of stenting, particularly of the overlapping case, and tube curvature on pressure drop inside the tube. Stenoses typically occur in curved arterial segments and bifurcations due to the strong relationship between the vascular atherogenesis process and recirculating zones induced by geometric conditions [39]. The statistically significant difference in $\overline{\overline{\Delta P}}$ between the control and overlapping stent groups suggests that both the presence of vessel curvature and the use of overlapping stents contribute to altering the hemodynamic behavior within the tube. This finding is consistent with previous studies indicating that stent overlap can create additional resistance or flow disturbances and as a result, blood flow and pressure profiles are affected [40]. Furthermore, the curvature of the vessel likely worsens these effects, as it may amplify the mechanical stress on the stent and the surrounding vessel walls, leading to higher pressure changes. In contrast, the absence of significant differences in pressure drop between the control and single stent groups suggests that single stent applications, regardless of whether the vessel is curved, do not influence the flow significantly.

### Relationships and Gender Differences

Of particular importance are also the relationships between the resulting hemodynamic and hemorheological factors derived in the study, specifically the effect of stenting on blood viscosity.

**Fig. 7:**
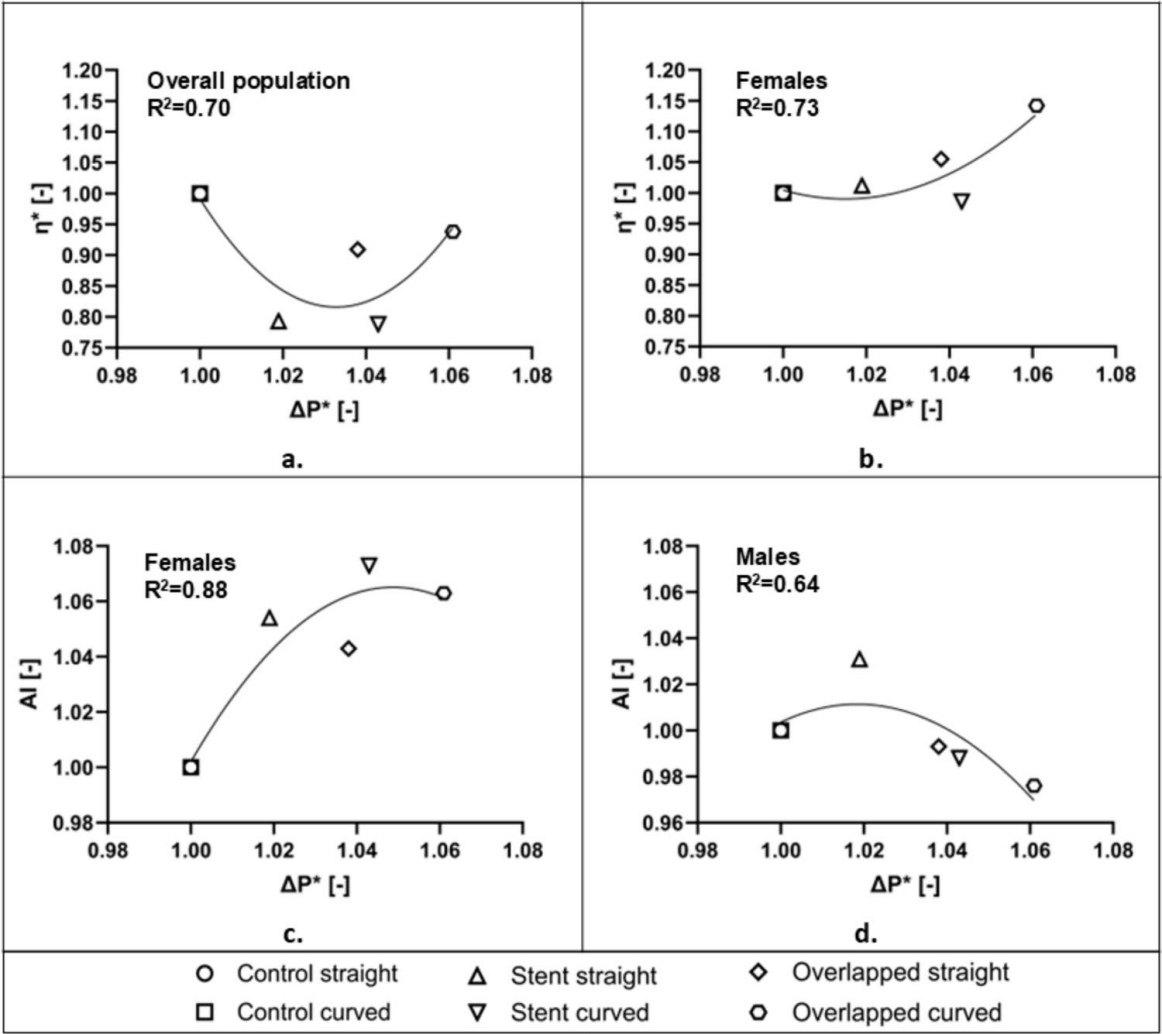
Correlation analysis. **a** Correlation between the stent-induced pressure-drop (ΔP*) and the measured viscosity (η*) of blood samples at a shear rate of 15.82 s⁻^1^ in the overall population. **b** Correlation between ΔP* and η* in the female group. **c** Correlation between ΔP* and aggregation index (AI) in females and **d** Correlation between ΔP* and AI in males

The overall effect of the stent-produced pressure drop on the measured viscosity of the blood samples is seen in the correlation graph of Fig. 7a. The pressure-drop ΔP* in this graph is the $\overline{\overline{\Delta P}}$ normalized in the same manner as the viscosity, i.e. the stented geometry $\overline{\overline{\Delta P}}$ values have been normalized with their corresponding control values, with unity implying the control cases. The viscosity values for the shear rate of 15.82 s^−1^ are used for the analysis. The graph in Fig. 7a indicates a non-linear relationship, with two distinct features: (a) an overall decreasing effect of the (stenting-produced) pressure drop, and (b) an increasing tendency of the viscosity as the pressure drop increases.

The relation of η* with ΔP* shows a complex behavior, as it is not in line with the monotonic increase expected for a Newtonian or non-Newtonian flow. Figure 7b illustrates a more clear relation between viscosity and pressure drop for the female population. This increasing tendency can be explained by considering the RBC aggregation behavior for the female samples seen in Fig. 4d, which is a statistically significant increasing trend against the stented configurations (p < 0.05, n = 4). RBC aggregation is the main determinant of the non-Newtonian nature of blood and is expected to affect negatively (i.e. increase) the effective viscosity of the fluid. This is in the absence of any slip effects (cell-free layers) that RBC aggregation produces, due to synaeresis or other migration phenomena [41]. Increased RBC aggregation in females compared to males is expected [42], however, the present results also show a sensitivity to the local hemodynamics, namely the resulting pressure-drop increase and stress exposure.

As differences in the female RBC aggregation (Fig. 4d) and overall pressure drop (Fig. 6), between control and stented configurations are statistically significant, the correlation of AI* (normalized AI with corresponding control values) against ΔP* is also of particular interest. Figure 7c shows that the increasing trend between AI* and ΔP* in the females, follows the general AI dependency on the stent configuration, seen in Fig. 4d. In contrast, the RBC aggregation behavior of the male population of the study (Fig. 7d) follows a different pattern: after an initial increase of AI* with ΔP*, a slight decrease follows.

The correlation coefficients seen in Fig. 7 indicate potential relationships between viscosity, RBC aggregation, and pressure drop. Perhaps of particular importance is the information extracted regarding the female population, as the study indicates a tendency of increasing viscosity as a result of the stent usage. This should be further investigated with more focused, and perhaps in vivo studies. As a general conclusion, it could be said that in clinical settings where minimizing pressure changes is crucial, single stent configurations may be preferred, particularly in cases where vessel curvature is not severe or complex.

## Conclusions

In this work, the influence of various stent configurations on the hematological, hemorheological and hemodynamical properties of blood was investigated, for two different flow conditions. In terms of hematology, the results showed no significant influence of the setups on RBC count, Hct, and MCV, with tendencies observed for exposure to more intense flow conditions. Regarding hemorheology effects, the results illustrated an influence on RBC aggregation and, to a lesser degree, on deformability, and hematocrit, which consequently may have affected the non-Newtonian behavior of the blood samples of the stented cases. Hemodynamic effects produced by the presence of stents were also revealed in the pressure measurements, as expected, with the overlapping stenting in the curved geometry being the most pronounced. Gender differences in RBC aggregation and viscosity effects were also revealed, which may aid in improving clinical practices. Further studies on local hemorheology and hemodynamics should contribute to a better understanding and refinement of the stenting practices.

## Data Availability

Not applicable.
